# Prolonged In Vitro Expansion Shapes the Neuro-Supportive Potential of Jaw Periosteum Secretomes: Implications for Secretome Product Quality

**DOI:** 10.3390/cells15171526

**Published:** 2026-08-24

**Authors:** Yuling Wang, Nuo Chen, Felix Umrath, Marina Danalache, Andreas Naros, Julia C. Fitzgerald, Dorothea Alexander

**Affiliations:** 1 Department of Oral and Maxillofacial Surgery, University Hospital Tübingen, 72076 Tübingen, Germany; yuling.wang1996@gmail.com (Y.W.); andreas.naros@med.uni-tuebingen.de (A.N.); 2 Laboratory of Cell Biology, Department of Orthopedic Surgery, University Hospital Tübingen, 72072 Tübingen, Germany; nuo.chen@med.uni-tuebingen.de (N.C.); marina.danalache@med.uni-tuebingen.de (M.D.); 3 Hertie Institute for Clinical Brain Research, University Hospital Tübingen, 72076 Tübingen, Germany; julia.fitzgerald@uni-tuebingen.de

**Keywords:** jaw periosteum, secretome, paracrine communication, passage-associated senescence-like phenotype, differentiation of iPSCs to NGN2 neurons, neuroregeneration

## Abstract

**Highlights:**

**What are the main findings?**

- Prolonged in vitro expansion alters the composition and biological activity of JPC-derived secretomes, resulting in reduced neuro-supportive capacity.
- Proteomic profiling demonstrates a remodeling of the JPC secretome, characterized by a shift from regenerative, extracellular matrix-related factors toward inflammatory and stress-associated signaling. Combined with metabolic activity kinetics and the detection of senescence-associated factors, we identified passage-associated cellular senescence as a key determinant of secretome quality.

**What are the implications of the main findings?**

- Passage-associated senescence status of JPCs should be considered a candidate critical quality attribute, as it directly influences secretome composition and regenerative potency.
- Monitoring and controlling cellular senescence during in vitro cell expansion may improve product consistency for a potential clinical translation of JPC-derived secretome therapeutics for nerve regeneration.

**Abstract:**

Nerve injuries are frequent complications of complex oral and maxillofacial surgical procedures, particularly following extensive tumor resections. Secretome-based, cell-free therapies derived from mesenchymal stromal cells have emerged as promising regenerative approaches; however, robust manufacturing requires the identification of critical quality attributes (CQAs) that ensure product potency and consistency. The influence of replicative senescence during in vitro expansion on the quality of jaw periosteum-derived mesenchymal stromal cell (JPC) secretomes has not yet been established. This study investigated whether the expansion state of JPCs affects the composition and neuro-supportive potency of their secretomes. Secretomes from four independent JPC donors were collected separately at early and late passages, pooled within each passage-specific preparation, and applied to human induced pluripotent stem cell-derived neurons. Neuronal survival, neurite outgrowth, and neuronal marker expression were assessed as functional readouts. Secretome composition was characterized by quantitative proteomics and enzyme-linked immunosorbent assay (ELISA) of selected senescence-associated secretory phenotype (SASP) factors. Secretomes derived from early-passage JPCs significantly enhanced neuronal survival and neurite outgrowth, whereas late-passage secretomes displayed reduced neuro-supportive activity. Proteomic profiling identified a pronounced shift toward inflammatory and stress-associated signaling, whereas performed ELISAs confirmed senescence-associated remodeling of the secretome, including increased abundance of SASP-associated factors in late-passage preparations. These findings demonstrate that prolonged in vitro expansion profoundly influences both the composition and biological potency of JPC-derived secretomes. Collectively, this study identifies the passage-associated senescence-like phenotype of JPCs as a key determinant of secretome quality and supports its consideration as a critical quality attribute for the manufacturing and standardization of JPC-derived secretome products. Monitoring and controlling the expansion state of JPCs may therefore be essential to ensure the consistency, potency, and clinical translation of secretome-based regenerative therapies.

## 1. Introduction

Nerve injury is a clinically relevant complication of oral and maxillofacial surgery, particularly involving the inferior alveolar and lingual nerves during procedures such as third molar extraction, implant placement, tumor resection, and orthognathic surgery [1]. Although surgical and biomaterial-based strategies can restore structural continuity and support tissue repair, functional recovery after nerve injury often remains incomplete [2,3,4]. Persistent sensory deficits, pain, and impaired neural function are partly attributed to the limited regenerative capacity of neurons and to a post-injury microenvironment characterized by inflammation, extracellular matrix (ECM) remodeling, and inhibitory signals that restrict neurite extension and functional reconnection [5,6,7]. Therefore, therapeutic strategies that support neuronal survival and neurite outgrowth are of increasing interest. Mesenchymal stem/stromal cells (MSCs) have been widely investigated for regenerative applications, but increasing evidence indicates that many of their therapeutic effects are mediated primarily through paracrine mechanisms rather than direct engraftment and differentiation [8,9,10]. The MSC secretome, consisting of soluble factors and extracellular vesicles, can regulate inflammation, apoptosis, angiogenesis, and endogenous repair processes [8,10,11]. In neural injury models, MSC-derived secretomes have been associated with improved neuronal survival, neurite extension, and functional recovery [12,13,14]. Compared with cell transplantation, secretome-based strategies offer advantages in terms of safety, standardization, storage, and clinical handling by reducing risks associated with immune incompatibility, uncontrolled differentiation, and tumorigenicity [10,15,16]. However, secretome composition and bioactivity are strongly influenced by cell source, culture conditions, expansion history, and cell physiological state [10,14,16]. These variables represent important considerations for the development of reproducible and clinically applicable cell-free products.

Jaw periosteum-derived mesenchymal stromal cells (JPCs) represent an MSC-like population obtained from the maxillofacial region, often during routine oral surgery with little donor-site morbidity [17,18]. Previous studies have shown that JPCs possess trilineage differentiation capacity and that their secretome can exert angiogenic and immunomodulatory effects under defined culture conditions [11,18,19]. However, whether prolonged in vitro expansion alters JPC-derived secretome composition and their neuro-supportive potential has not yet been investigated.

In the present study, we compared early- and late-passage JPCs, assessed senescence-associated cellular and secretory changes, performed comparative proteomic profiling of JPC-derived secretomes, and evaluated their effects on survival, neurite outgrowth, and marker expression of iPSC-derived NGN2-induced neurons. The study was designed to determine whether source-cell expansion state of the producer cells, together with secretome composition, represents a critical quality attribute that should be monitored during the manufacturing and standardization of JPC-derived secretome therapeutics for neural repair.

## 2. Materials and Methods

### 2.1. Cell Culture

JPCs were isolated from four healthy donors (two males and two females, aged 20–31 years) after written informed consent. The study was approved by the local ethics committee (Approval Nos. 618/2017BO2 and 358/2025BO2). JPCs were cultured in DMEM/F-12 (Gibco, Thermo Fisher Scientific, Waltham, MA, USA) supplemented with 10% human platelet lysate (hPL; PL BioScience, Aachen, Germany), 100 U/mL penicillin-streptomycin (P/S; Gibco), and 2.5 µg/mL amphotericin B (neoFroxx, Einhausen, Germany). Passage 5 (P5) was selected to represent an earlier expansion state because JPCs at this passage retained trilineage differentiation capacity in the present study, consistent with previous studies from our group demonstrating differentiation potential of JPCs around passages 5–6 [20]. Passage 10 (P10) was selected as a more extensively expanded state to investigate functional and senescence-associated alterations resulting from prolonged in vitro culture. Accordingly, P5 and P10 were used as representative earlier and more extensively expanded states, respectively, for all subsequent experiments.

Induced pluripotent stem cells (iPSCs) were generated in our lab from JPCs obtained from a healthy 29-year-old female donor undergoing wisdom tooth extraction, as previously described [21]. The cells displayed a normal karyotype and tested negative for mycoplasma. JPC-derived iPSCs were maintained on 6-well plates coated with 1% Matrigel (Corning Inc., Corning, NY, USA) in mTeSR™ Plus medium (STEMCELL Technologies, Vancouver, BC, Canada). Cells between passages 10 and 30 were used for neuronal induction.

### 2.2. Trilineage Differentiation Assays

The multilineage potential of passage 5 JPCs was assessed by osteogenic, adipogenic, and chondrogenic induction using established protocols [22,23,24]. After 21 days of induction, osteogenic, adipogenic, and chondrogenic differentiation were evaluated by Alizarin Red S, Oil Red O, and Toluidine Blue staining, respectively. Detailed induction conditions are provided in the Appendix A Section.

### 2.3. Generation of NGN2-Induced Excitatory Neurons

NGN2-induced excitatory neurons were generated from human iPSCs using doxycycline-inducible neurogenin-2 (NGN2) expression based on a modified protocol [25]. Briefly, JPC-derived iPSCs were transduced with pLV-TetO-hNGN2-GFP-Puro and FUdeltaGW-rtTA lentiviral vectors provided by the collaborating laboratory headed by Dr. Julia Fitzgerald’s. NGN2 expression was induced with doxycycline, followed by puromycin selection. On day 4, cells were transferred to Neurobasal medium (Gibco) supplemented with 1× GlutaMAX, 1× non-essential amino acids (NEAA; Gibco), 1× N2 supplement (Gibco), 1× B-27™ Supplement (Gibco), 10 ng/mL BDNF (PeproTech, Cranbury, NJ, USA) and 1 µg/mL doxycycline (Selleck Chemicals, Houston, TX, USA). NGN2-induced neurons were used directly or cryopreserved at day 4 for later downstream experiments. Detailed induction conditions are provided in the Appendix A Section.

### 2.4. Assessment of Cellular Metabolic Activity

Metabolic activities of JPCs were evaluated using the Cell Counting Kit-8 (CCK-8) assay (Selleck Chemicals, Houston, TX, USA). JPCs were seeded in 96-well plates at 2000 cells per well. At 24, 48, 72, and 96 h, CCK-8 reagent was added and absorbance at 450 nm was measured using a microplate reader (BioTek Instruments, Winooski, VT, USA). Changes in CCK-8 signal were calculated as an indicator of metabolic activity/viability. To compare the metabolic activity among groups during exponential growth, the relative metabolic activity µ was calculated from CCK-8 absorbance values using the following formula:

$$\mu \left[\frac{1}{h}\right]=\frac{\ln\left(\frac{OD_{96h}}{OD_{48h}}\right)}{96h-48h}$$

where OD_48h_ and OD_96h_ represent the absorbance values measured at 48 and 96 h, respectively.

### 2.5. Quantification of Cellular Reactive Oxygen Species and Senescence-Associated β-Galactosidase

Intracellular reactive oxygen species (ROS) were quantified using a DCFDA-based assay (Abcam, Cambridge, UK) at day 4, as used for secretome collection. JPCs were incubated with 20 µM DCFDA for 30 min at 37 °C and analyzed using a Guava easyCyte 6HT-2L flow cytometer (Merck Millipore, Billerica, MA, USA). Senescence-associated β-galactosidase activity was assessed (at day 4, as used for secretome collection) using SPiDER-β-Gal (Dojindo Molecular Technologies, Kumamoto, Japan) according to the manufacturer’s instructions and quantified by flow cytometry. For both assays, debris was excluded based on forward and side scatter, and mean fluorescence intensity was used for quantification.

### 2.6. Secretome Collection and Enrichment

The collected conditioned medium, hereafter referred to as the JPC secretome, was collected from P5 and P10 JPCs using a previously described serum-free conditioning and ultrafiltration-based procedure with minor modifications [26]. For secretome collection, JPCs from four donors were seeded separately into T175 flasks at 2 × 10^6^ cells per flask and cultured until reaching 80–90% confluence. At day 4 after cell seeding, cells were then washed three times with DPBS and once with basal medium (DMEM/F-12 supplemented with 100 U/mL penicillin-streptomycin and 2.5 µg/mL amphotericin B) and incubated in 37 mL fresh basal medium for 24 h. Conditioned medium was collected, centrifuged at 600× *g* for 7 min at 4 °C to remove cell debris, and the 34 mL supernatant was snap-frozen in liquid nitrogen and stored at −80 °C. Secretome samples were concentrated approximately 100-fold using Vivaspin 20 centrifugal concentrators (5 kDa MWCO; Sartorius, Göttingen, Germany). The retained fractions from four donors were pooled within each passage-specific preparation, aliquoted, and stored at −80 °C until use.

### 2.7. Protein Quantification and ELISA Analysis of Secretome Samples

Total protein concentration was determined using a BCA Protein Assay Kit (Thermo Fisher Scientific, Waltham, MA, USA). Concentrations of IL-6, IL-8, CCL2, CCL20, TGF-β1, and VEGF in the retained fractions were measured using commercially available ELISA kits (R&D Systems, Bio-Techne, Wiesbaden, Germany) according to the manufacturers’ protocols. Quantitative analyses of the aforementioned factors were performed for three aliquots of pooled-secretome preparations per passage condition. Target protein concentrations were normalized to the total protein concentration of the corresponding secretome sample.

### 2.8. Proteomic Analysis of Secretome Samples

Secretome samples were analyzed by a proteomics core facility using four aliquots of the pooled-secretome preparation per passage condition. These aliquots represented technical replicates for each passage condition. The technical replicates were retained as separate measurements in the statistical analysis to characterize technical variability within each pooled-secretome preparation. Accordingly, statistical significance and adjusted *p* values reflect differences between the two pooled-secretome preparations relative to technical measurement variability and do not represent donor-level biological variability. We did not consider a cell-free medium control for proteomic analyses because the medium used (DMEM/F-12) contains no proteins but only the essential amino acids. After concentration using the 5 kDa molecular-weight-cutoff, the proteins are retained but neither GlutaMAx nor amino acids are retained. Therefore, there are no proteins that would interfere with downstream proteomic analyses. Protein intensity data were processed using Perseus (v2.1.3.0; Max Planck Institute of Biochemistry, Martinsried, Germany). After log2 transformation and quantile normalization, differentially expressed proteins (DEPs) were identified using an adjusted *p* value < 0.05 and an absolute log2 Fold Change (|log2FC|) ≥ 1. Principal component analysis, hierarchical clustering, heatmap visualization, and Gene Ontology and Kyoto Encyclopedia of Genes and Genomes enrichment analyses were performed in R (v4.4.1; R Foundation for Statistical Computing, Vienna, Austria).

### 2.9. Treatment of NGN2-Induced Neurons with JPC Secretomes

NGN2-induced neurons generated from a single transduction batch were cryopreserved and used in three independently conducted thaw-and-treatment experiments. Cells were seeded in Matrigel-coated 24-well plates at 5 × 10^4^ cells per well. After 24 h, cultures were assigned to four groups: negative control, positive control, JPC P5 secretome, and JPC P10 secretome. The negative control consisted of Neurobasal medium containing 1× GlutaMAX and 1× NEAA, and all other treatment media were based on this formulation. The positive control medium was further supplemented with 1× N2 supplement, 1× B-27™ Supplement, and 10 ng/mL BDNF, whereas the JPC P5 and JPC P10 groups received 500 µg/mL of pooled secretome (total protein) derived from passage 5 or passage 10 JPCs, respectively. Half-medium changes were performed every 2 days for 6 days.

### 2.10. Immunofluorescence Staining and Image Analysis

Immunofluorescence staining was performed to assess neuronal differentiation and neurite outgrowth. Cells were fixed, permeabilized, blocked, and incubated with primary antibodies against doublecortin (DCX; mouse, 1:1000; Abcam) and microtubule-associated protein 2 (MAP2; rabbit, 1:200; Abcam), followed by Alexa Fluor 488-conjugated goat anti-mouse IgG and Alexa Fluor 594-conjugated goat anti-rabbit IgG secondary antibodies (both 1:1000; Abcam). Nuclei were counterstained with DAPI (1 µg/mL; BioLegend, San Diego, CA, USA), and images were acquired using an Axio Observer Z1 fluorescence microscope (Zeiss, Jena, Germany).

Images were analyzed using ImageJ 1.54p (NIH, Bethesda, MD, USA). Across the three independently conducted thaw-and-treatment experiments, in total 9–10 replicates (wells) per condition were included for the final analysis. Nine images were acquired per well under identical microscope settings and averaged to obtain one well-level value. MAP2 (microtubule-associated protein-2)-positive cells and DAPI-stained nuclei were quantified per field of view as indicators of neuronal differentiation and overall cell survival, respectively. Neurite outgrowth was quantified in DCX-stained cells using the Ridge Detection plugin, and total neurite length per field of view was extracted. Identical image-processing parameters were applied across all conditions.

### 2.11. Quantitative Gene Expression Analysis of JPCs

Total RNA was isolated from adherent JPCs (*n* = 4 secretome donors) at day 4 after cell seeding and reverse-transcribed into cDNA. Quantitative real-time PCR (qPCR) was performed using PowerTrack™ SYBR Green Master Mix (Applied Biosystems™, Thermo Fisher Scientific, Waltham, MA, USA) and primers for GAPDH, IL6, IL8, CCL2, CCL20, VEGFA, TGFB1, CDKN2A, CDKN1A, TP53, NGF, and GDNF. Primer sequences are provided in Appendix A, Table A1. Relative gene expression was calculated using the 2^−ΔΔCt^ method with GAPDH as the reference gene.

### 2.12. Statistical Analysis

Statistical analyses were performed using GraphPad Prism (v8.0.2; GraphPad Software, Boston, MA, USA). Data are presented as mean ± standard deviation (SD). ROS, SA-β-gal activity, ELISA measurements, and qPCR results were analyzed using unpaired Student’s *t*-test. CCK-8-derived metabolic activity was analyzed by two-way analysis of variance (ANOVA) with the factors group (P5 vs. P10) and time (24, 48, 72, and 96 h); where appropriate, simple main effects at 96 h were examined. Immunofluorescence quantification was analyzed using Welch’s one-way ANOVA followed by the Games–Howell post hoc test. Statistical significance was defined as *p* < 0.05.

## 3. Results

**Figure 1:**
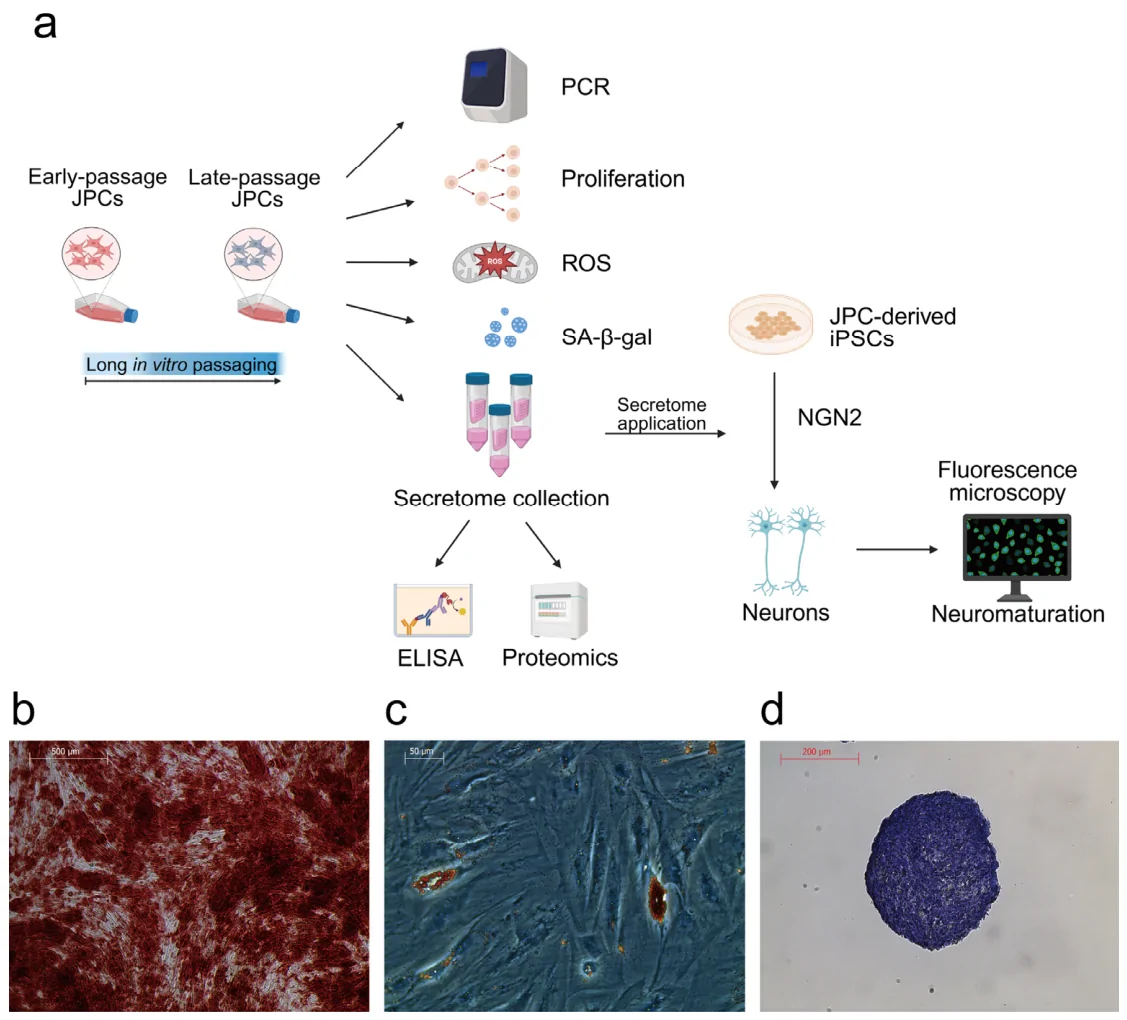
Experimental workflow and trilineage differentiation potential of JPCs. (**a**) Experimental workflow for evaluating passage-associated changes in JPCs and the neuro-supportive effects of JPC-derived secretomes. Early- and late-passage JPCs were generated by long-term in vitro passaging. Senescence-associated phenotypes were assessed by gene expression analysis, assessment of metabolic activity using the CCK-8 assay, intracellular ROS measurement, and SA-β-gal activity. Collected secretomes were characterized by ELISA and proteomic analysis. JPC-derived iPSCs were differentiated into neurons by neurogenin-2 (NGN2) transduction and treated with JPC-derived secretomes, followed by fluorescence-based endpoint analysis. Created with BioRender.com (https://BioRender.com/a9jfgrg). (**b**–**d**) Representative images showing trilineage differentiation of passage 5 JPCs after 21 days of induction. (**b**) Osteogenic differentiation assessed by Alizarin Red S staining showing mineralized matrix deposition; scale bar = 500 µm. (**c**) Adipogenic differentiation assessed by Oil Red O staining showing intracellular lipid droplets; scale bar = 50 µm. (**d**) Chondrogenic differentiation assessed by Toluidine Blue staining showing proteoglycan-rich extracellular matrix deposition; scale bar = 200 µm.

Figure 1a gives an overview of the experimental set-up for the present study.

### 3.1. Trilineage Differentiation of JPCs

To confirm the mesenchymal stromal phenotype of JPCs, passage 5 cells were subjected to trilineage differentiation. After 21 days, JPCs showed mineralized matrix deposition by Alizarin Red S staining, intracellular lipid accumulation by Oil Red O staining, and proteoglycan-rich matrix deposition by Toluidine Blue staining under osteogenic, adipogenic, and chondrogenic conditions, respectively (Figure 1b–d). These findings confirmed the trilineage differentiation potential of used JPCs for secretome collection.

### 3.2. Early-Passage JPCs Show Enhanced Metabolic Activities, Reduced ROS Levels, SA-β-Gal Activity and p16 mRNA Levels Compared to Late-Passage JPCs

**Figure 2:**
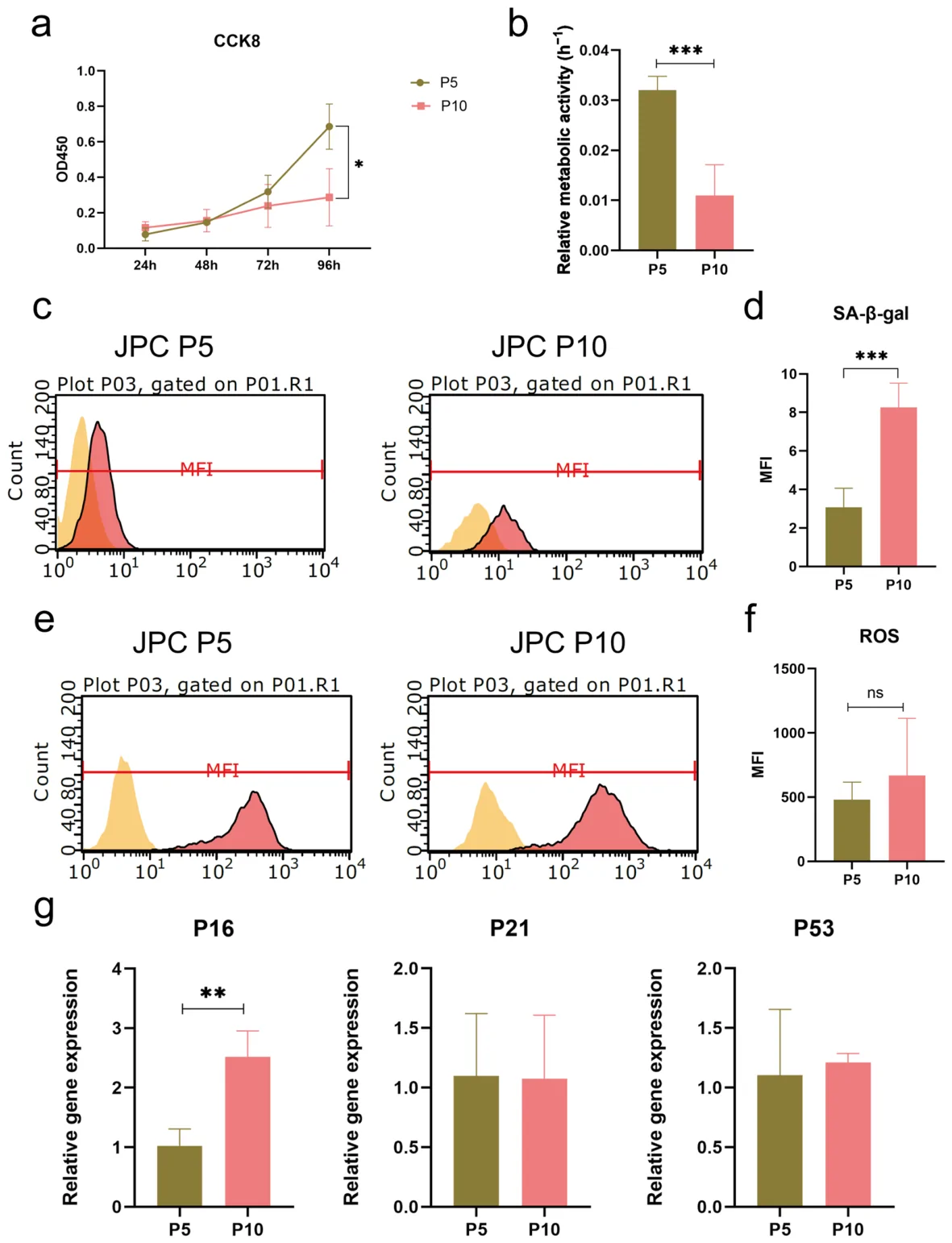
Metabolic activity and senescence-associated changes in early- and late-passage JPCs. (**a**) Metabolic curves of passage 5 (P5) and passage 10 (P10) JPCs measured at 24, 48, 72, and 96 h using the CCK-8 assay with a significant difference at 96 h (*p* = 0.0352). (**b**) Quantification of OD450 values obtained by CCK-8 measurements during the exponential phase 48–96 h in P5 than in P10 cells, with a significant difference (*p* = 0.0008). (**c**) Representative flow cytometry histograms of senescence-associated β-galactosidase (SA-β-gal) fluorescence in P5 and P10 JPCs. (**d**) Quantification of SA-β-gal mean fluorescence intensity (MFI), showing significantly higher levels in P10 cells (*n* = 4 donors per passage, *p* = 0.0007). (**e**) Representative flow cytometry histograms of intracellular reactive oxygen species (ROS) levels in P5 and P10 JPCs. (**f**) Quantification of ROS MFI, showing a slight but non-significant increase in P10 cells (*n* = 4 donors per passage). (**g**) Quantitative real-time PCR analysis of senescence-associated genes in P5 and P10 JPCs (*n* = 4 independent secretome donors per passage). Relative expression levels were normalized to GAPDH and calculated using the 2^−ΔΔCt^ method. CDKN2A (p16) expression was significantly increased in P10 cells (*p* = 0.0012). Data are presented as mean ± SD. Statistical analysis was performed using two-way ANOVA for the proliferation time-course data, with 96 h comparison shown in (**b**), and unpaired two-tailed Student’s *t*-tests for (**d**,**f**,**g**). ns, not significant; * *p* < 0.05; ** *p* < 0.01; *** *p* < 0.001.

To determine whether prolonged in vitro expansion affects JPC metabolic activity and senescence-associated features, P5 and P10 cells were compared. CCK-8 analysis showed comparable metabolic activities at early time points, whereas P5 cells displayed higher metabolic activity from 72 h onward, reaching significance at 96 h (*p* = 0.0352; Figure 2a). Consistent with this finding, the CCK-8-derived relative metabolic activity calculated over the exponential 48–96 h interval was significantly higher in P5 than in P10 cells (*p* = 0.0008; Figure 2b). P10 JPCs exhibited significantly increased SA-β-gal activities compared with P5 cells (*p* = 0.0007; Figure 2c,d), indicating an enhanced passage-associated senescence-like phenotype. ROS levels showed only a slight, nonsignificant increase in P10 cells (Figure 2e,f), suggesting that oxidative stress was not substantially elevated under these conditions.

**Figure A2:**
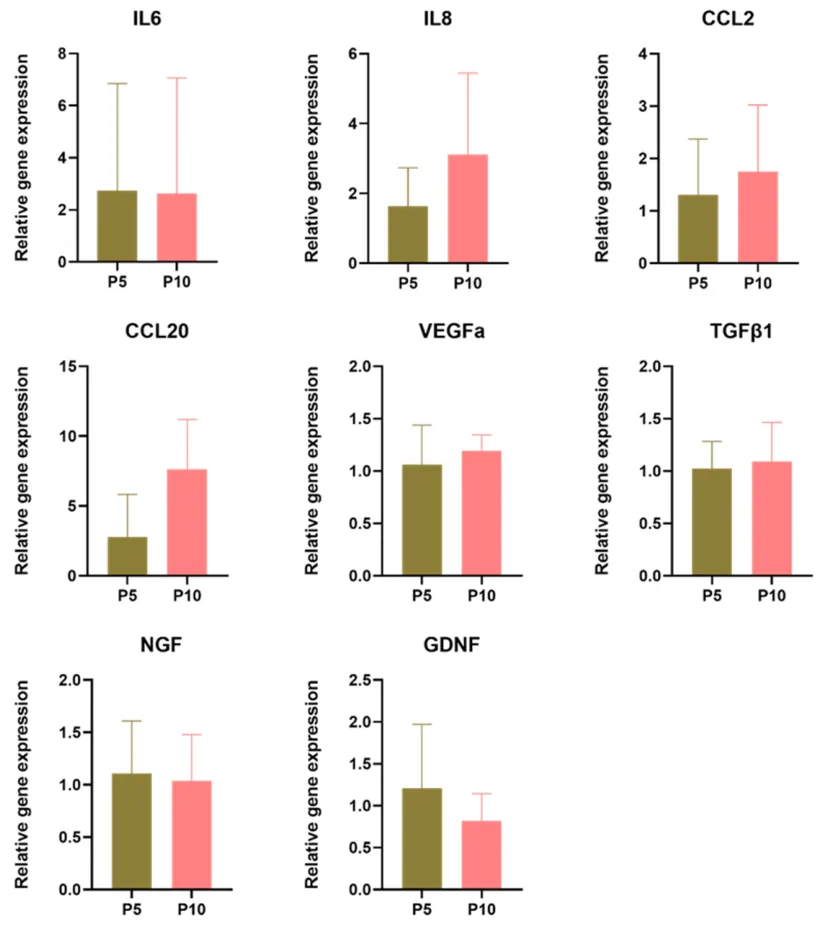
Additional transcriptional changes in early- and late-passage JPCs. qPCR analysis of SASP-related genes and neurotrophic factors in passage 5 (P5) and passage 10 (P10) JPCs. Relative expression levels of IL6, IL8, CCL2, CCL20, VEGFA, TGFB1, NGF, and GDNF were normalized to GAPDH and calculated using the 2^−ΔΔCt^ method. Data are presented as mean ± SD (*n* = 4). Statistical analysis was performed using unpaired two-tailed Student’s *t*-tests.

Quantitative PCR analysis further supported a senescence-like phenotype in late-passage JPCs. CDKN2A (p16) expression was significantly increased in P10 cells (*p* = 0.0012), whereas CDKN1A (p21) and TP53 remained unchanged (Figure 2g). Additional qPCR analysis showed non-significant upward trends in SASP-related transcripts and a tendency toward reduced NGF and GDNF expression in P10 cells compared with P5 cells (Appendix A, Figure A2).

### 3.3. Increased SASP Factor Concentration in P10 JPC Secretome

**Figure 3:**
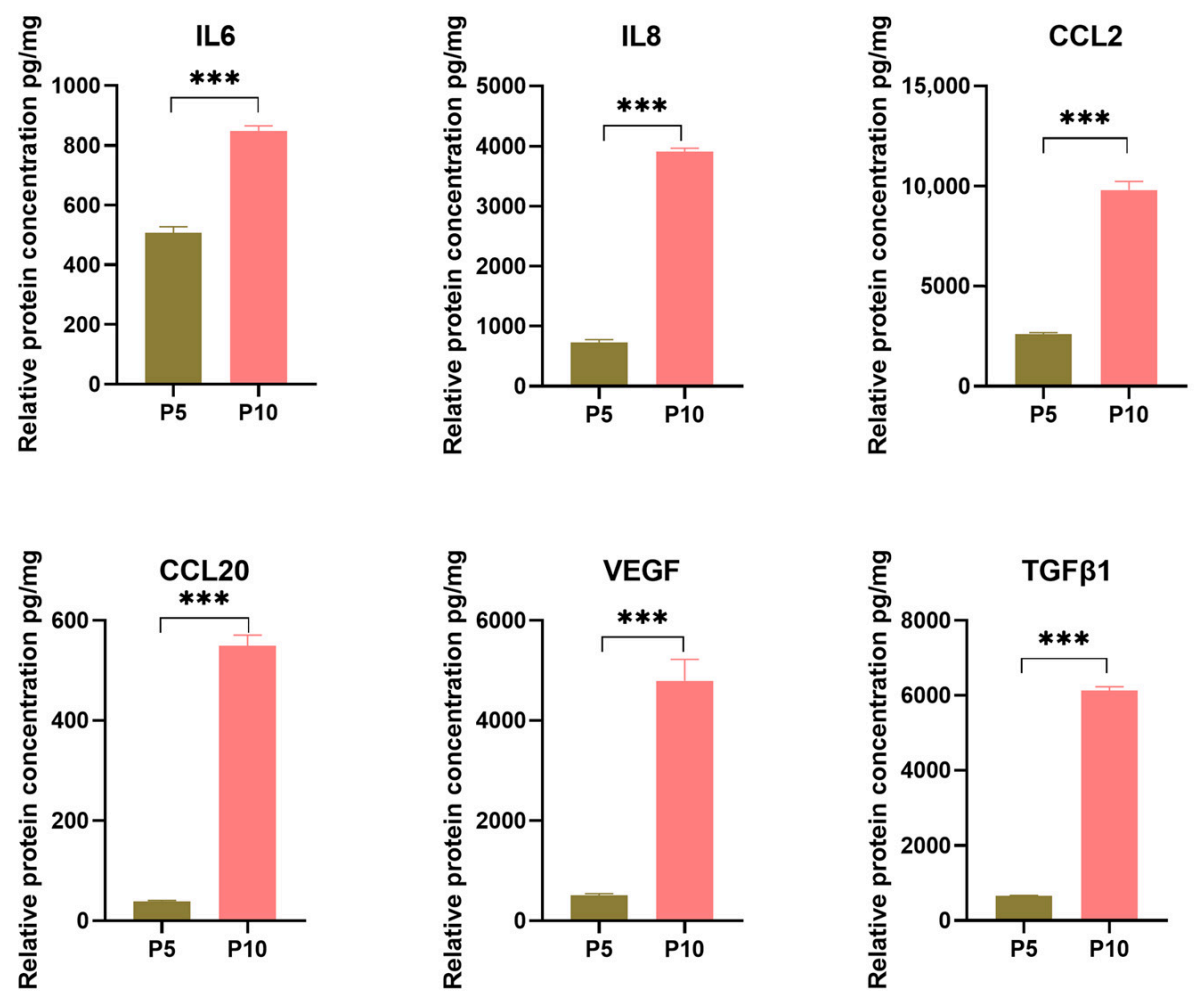
Quantification of senescence-associated secretory phenotype (SASP) factors in early- and late-passage JPC secretomes. ELISA quantification of IL-6, IL-8, CCL2, CCL20, VEGF, and TGF-β1 in P5 and P10 JPC secretomes. All measured SASP-associated factors were elevated in P10 secretomes compared with P5 secretomes (*p* < 0.001). Data are presented as mean ± SD (*n* = 3 aliquots from the pooled secretome). Statistical analysis was performed using unpaired two-tailed Student’s *t*-tests. *** *p* < 0.001.

To assess passage-dependent changes in the JPC secretome, selected SASP-associated proteins were quantified by ELISA and normalized to total secretome protein. Compared with P5 secretomes, P10 secretomes showed a broad and significant increase in IL-6, IL-8, CCL2, CCL20, VEGF, and TGF-β1 levels (all *p* < 0.0001; Figure 3). These findings indicate that P10 JPC secretomes acquire a more pronounced SASP-associated profile than the P5 JPC secretomes.

### 3.4. Proteomic Profiling Reveals Distinct Signatures Between P5 and P10 JPC Secretomes

**Figure 4:**
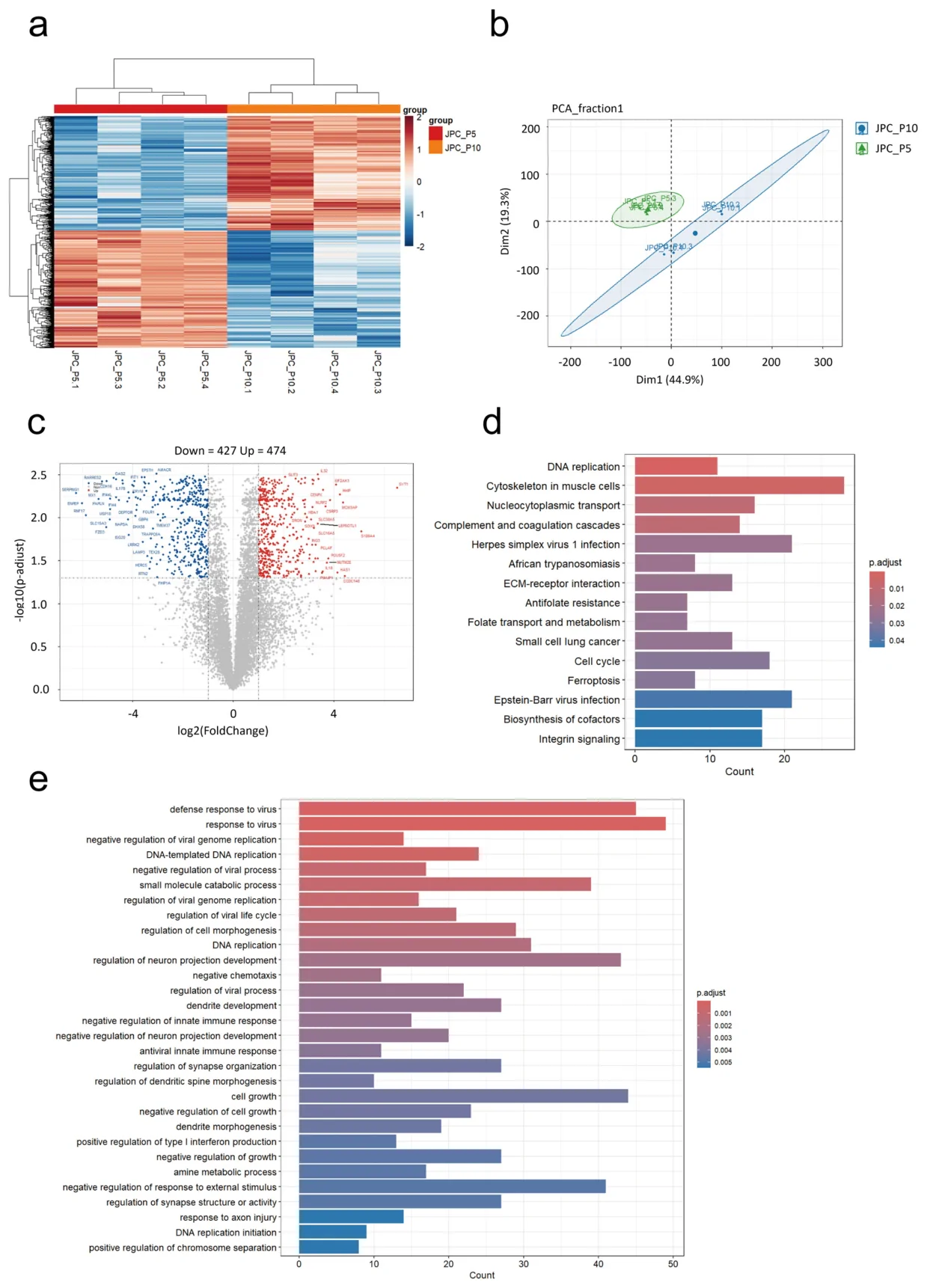

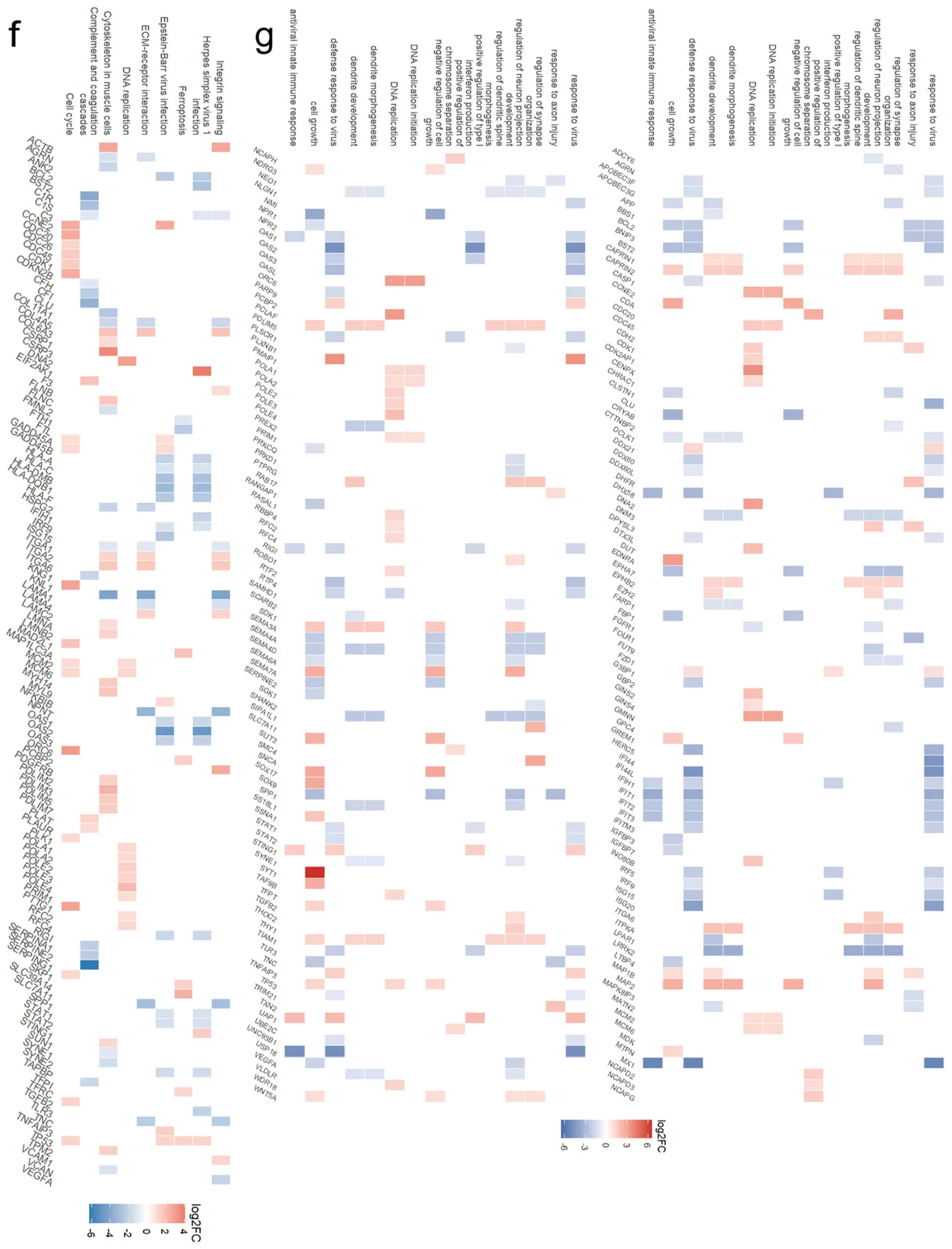
Proteomic profiling and functional enrichment analysis of secretomes from early- and late-passage JPCs. (**a**) Heatmap with hierarchical clustering of differentially expressed proteins (DEPs) between passage 5 (P5) and passage 10 (P10) JPC secretomes. Expression values were scaled by Z-score. (**b**) Principal component analysis (PCA) showing separation of P5 and P10 samples; ellipses indicate 95% confidence intervals (green P5, blue P10 JPCs). (**c**) Volcano plot of DEPs between P5 and P10 secretomes. Proteins with |log2FC| ≥ 1 and adjusted *p* < 0.05 are highlighted. (**d**) Significantly enriched KEGG pathways based on DEPs. (**e**) Heatmap of log2FC values for proteins associated with enriched KEGG pathways. (**f**) Top 30 significantly enriched Gene Ontology (GO) terms based on DEPs. (**g**) Heatmap of log2FC values for proteins associated with enriched GO terms. In (**a**), red and blue indicate relatively higher and lower expression, respectively. In (**c**), red and blue indicate proteins upregulated and downregulated in P5 secretomes versus P10 secretomes, respectively. In (**f**,**g**), red and blue indicate upregulation and downregulation, respectively, in P5 secretomes relative to P10 secretomes. The four samples per passage condition represent technical replicates derived from a single pooled-secretome preparation.

Label-free quantitative proteomics was performed to characterize global secretome differences between pooled P5 and P10 JPC secretomes. In total, 9365 proteins were identified and quantified with high reproducibility across four technical replicates derived from one pooled-secretome preparation per passage condition. Principal component analysis showed clear separation between P5 and P10 secretomes, indicating distinct proteomic profiles (Figure 4a,b). Using an adjusted *p* value < 0.05 and |log2FC| ≥ 1, 901 differentially expressed proteins were identified, including 474 proteins with higher abundance and 427 proteins with lower abundance in P5 relative to P10 secretomes (Figure 4c). For presentation of the most strongly altered proteins in Appendix A, Table A2, a more stringent secondary threshold of |log2FC| ≥ 4 was applied.

Functional enrichment analysis showed that passage-dependent secretome remodeling involved cellular aging, inflammatory and immune responses, extracellular matrix interactions, and neuronal development. KEGG analysis highlighted cell cycle, DNA replication, ferroptosis, complement and coagulation cascades, ECM-receptor interaction, integrin signaling, and cytoskeleton-associated pathways (Figure 4d,f). GO analysis further identified immune and antiviral responses, neuron projection development, dendrite morphogenesis, synapse organization, and response to axon injury (Figure 4e,g). Representative P5-enriched proteins included SYT1, S100A4, HHIP, and HAS1, whereas interferon- and antiviral response-associated proteins, including ISG20, IFI44L, OAS2, USP18, and MX1, were enriched in P10 secretomes. Additional ECM- and adhesion-related proteins, including ITGA2, ITGA6, COL6A3, and LAMC2, were found to be more abundant in P5 secretomes but did not meet the stringent |log2FC| ≥ 4 cutoff. The complete proteomic dataset, including differentially abundant proteins and enrichment results, is provided in Table S1. Highly regulated proteins meeting this stringent cutoff are listed in Table A2 included in the Appendix A Section.

### 3.5. Effects of JPC Secretomes on NGN2-Induced Neurons

**Figure 5:**
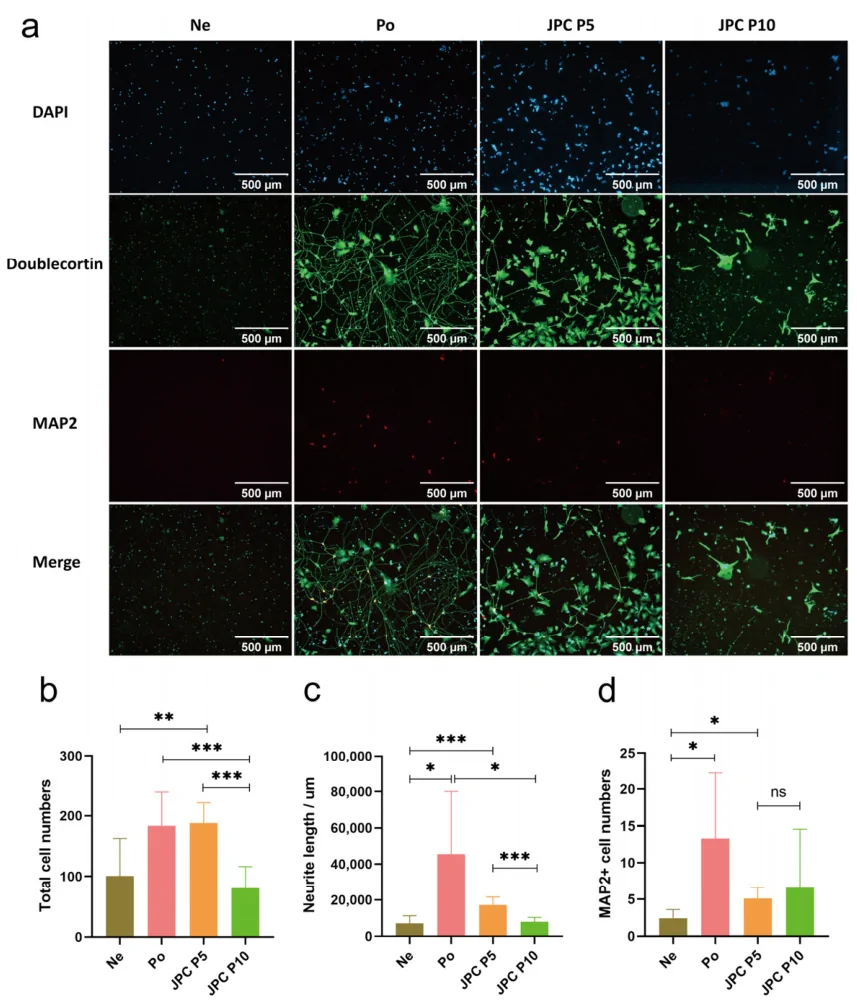
Effects of JPC secretomes from different passages on survival, neurite outgrowth, and MAP2-associated maturation of induced neurons. (**a**) Representative immunofluorescence images of induced neurons cultured under negative control (Ne), positive control (Po), JPC P5 secretome, and JPC P10 secretome conditions. Nuclei were stained with DAPI (blue), immature neurons with doublecortin (DCX, green), and MAP2-positive neurons with MAP2 (red). Merged images are shown; scale bar = 500 μm. (**b**) Quantification of cell survival based on the number of DAPI-positive nuclei per field of view. (**c**) Quantification of neurite outgrowth, expressed as total neurite length per field of view. (**d**) Quantification of MAP2-positive cells per field of view as an indicator of neuronal maturation. Data are presented as mean ± SD from three independently generated thaw-and-treatment experiment series with 3–4 replicates per series, so in total 9–10 replicates were visualized and quantified per condition (Ne, *n* = 10; Po, *n* = 10; JPC P5, *n* = 10; JPC P10, *n* = 9). Statistical analysis was performed using Welch’s one-way ANOVA followed by the Games-Howell post hoc test. ns, not significant; * *p* < 0.05; ** *p* < 0.01; *** *p* < 0.001.

To evaluate functional consequences of passage-dependent secretome remodeling, NGN2-induced neurons (once transduction batch was used for 3 independent thaw-and-treatment experiment series) were treated with P5 or P10 JPC secretomes (in triplicate for every series). Quantification was performed per field of view under identical imaging conditions (Figure 5a). P5 secretome significantly increased total cell number compared with the negative control (*p* = 0.005) and P10 secretome (*p* < 0.001), reaching levels comparable to the positive control (Figure 5b). P5 secretome also significantly enhanced neurite outgrowth compared with both the negative control and P10 secretome (both *p* < 0.001; Figure 5c). MAP2-positive cells were shown to be significantly increased by P5 secretome compared with the negative control (*p* = 0.002), indicating enhanced neuronal maturation-associated marker expression (Figure 5d). The P10 secretome showed intermediate effects and only in the tendency an increase in MAP2 cell numbers, and no significant difference was detected between the P5 and P10 groups.

## 4. Discussion

Replicative senescence is increasingly recognized as a critical determinant of MSC function because it alters secretome composition and, consequently, paracrine potency. In the context of developing MSC-derived secretome therapeutics, characterization of source-cell senescence is therefore highly relevant for defining product quality. In the present study, prolonged in vitro expansion induces a passage-associated senescence-like phenotype in JPCs that was accompanied by marked remodeling of the secretome and diminished neuro-supportive activity, demonstrating that the biological potency of JPC-derived secretomes is strongly influenced by the expansion state and culture history of the producer cells [27,28].

Late-passage JPCs exhibited significantly reduced metabolic activities, increased SA-β-gal activity, and marked upregulation of CDKN2A (p16), whereas CDKN1A (p21) and TP53 remained unchanged. This expression pattern is consistent with a predominantly p16-associated senescence program rather than pronounced activation of the p53/p21 pathway under the conditions examined, although it does not establish irreversible cell-cycle arrest or a stable late-stage senescent phenotype [29,30]. Mesenchymal stromal cell senescence is characterized by secretion of IL-6, IL-8, CCL2, CCL20, TGF-β1 and VEGF, among others [27] and we detected all these SASP factors to be significantly elevated in P10 JPCs. Intracellular ROS levels were not significantly different between P5 and P10 cells, suggesting that the observed phenotype was not primarily driven by acute oxidative stress. This distinction is important because replicative aging and stress-induced premature senescence can generate overlapping yet functionally distinct secretory phenotypes that may differently affect the quality and potency of secretome-based products. Additional analyses of cell-cycle status, DNA damage, and reversibility would be required to discriminate these senescence states more definitively [31,32,33,34].

The passage-associated cellular alterations were accompanied by pronounced remodeling of the JPC secretome, indicating that senescence-related changes in producer cells are directly reflected in the composition of the therapeutic product. Increased abundance of SASP-associated proteins in P10 secretomes suggested a shift toward a more inflammatory secretory phenotype [35]. Proteomic profiling further revealed a transition from extracellular matrix (ECM), adhesion, and neurodevelopment-associated proteins toward interferon-, immune-, complement-, and stress-related signaling. Proteins enriched in P5 secretomes, including SYT1, S100A4, HHIP, and HAS1, are associated with synaptic function, cytoskeletal organization, developmental signaling, and hyaluronan-mediated matrix remodeling [36,37,38], while ECM- and adhesion-related proteins such as ITGA2, ITGA6, COL6A3, and LAMC2 were likewise more abundant [38,39]. In contrast, P10 secretomes were enriched in interferon- and antiviral response proteins (ISG20, IFI44, IFI44L, OAS2, MX1, and USP18), together with immune-regulatory, complement-associated, and matrix-remodeling proteins including IL17B, RARRES2, SERPING1, and TNFAIP6 [40,41,42]. Although several proteins enriched in P10-secretomes, including FZD3, ALDH1A1, SOD3, LAMA1, and PAPLN, possess developmental, antioxidant, or matrix-related functions [43,44], they were embedded within a broader inflammatory and stress-associated secretome landscape that did not translate into improved neuronal responses. These observations indicate that secretome potency is determined by the integrated molecular composition rather than by the presence of individual bioactive proteins, highlighting the importance of comprehensive molecular characterization as part of quality assessment.

We have to state that the detected proteins reflect not only the normal secretory state but also the cells’ response to growth factor deprivation. Therefore, careful standardization of starvation conditions (duration, residual serum/platelet lysate, medium) should be taken into account for future development of next-generation therapeutic products. Other factors such as cell type or pre-/post-treatment can influence the output as well. Giannasi and colleagues showed that a 72-h serum deprivation of adipose-derived MSCs does not induce changes in cell phenotype while it affects cell metabolism by modifying mitochondrial activity and inducing oxidative stress [45]. On the other hand, Piccolo and co-authors demonstrated that secretomes from adipose-derived MSCs, firstly stimulated with IL-1β and then subjected to serum starvation for 24 h, contained fewer pro-inflammatory mediators, partially restored their regenerative profile, and elicited fewer pro-inflammatory effects on osteoarthritis chondrocytes than before starvation [46].

The proteomic differences were reflected in the functional assays. Early-passage secretomes more effectively promoted neuronal survival and neurite extension than late-passage secretomes. Likewise, P5 secretomes significantly increased the number of MAP2-positive neurons compared with the negative control, whereas P10 secretomes produced only a non-significant trend. These findings suggest that neuronal survival and neurite outgrowth are particularly sensitive to senescence-associated alterations in ECM organization, adhesion signaling, cytoskeletal regulation, and inflammatory pathways, whereas neuronal differentiation, as assessed by MAP2 expression, appears comparatively less affected. Nevertheless, the present study identifies candidate mechanisms rather than establishing causal relationships between individual secretome components and functional outcomes.

Previous studies have demonstrated that MSC-derived secretomes promote neuronal viability, neurite outgrowth, axonal regeneration, and functional recovery through soluble trophic factors, extracellular vesicles, ECM-associated proteins, and immunomodulatory mediators [12,13]. However, direct comparisons between studies remain challenging because conditioning protocols, concentration procedures, molecular weight cut-offs, normalization strategies, and dosing differ substantially. In our study, the superior biological activity of the P5 secretome is consistent with its enrichment in ECM- and adhesion-associated proteins known to regulate neuronal attachment, growth-cone dynamics, cytoskeletal remodeling, and axonal extension, whereas inflammatory and stress-associated mediators can adversely influence neuronal regenerative responses [37,38,47,48]. Collectively, these data indicate that the neuro-supportive potency of JPC-derived secretomes is closely linked to the molecular composition established during source-cell expansion.

From a translational and manufacturing perspective, these findings identify the expansion state of producer cells and the resulting secretome composition as critical quality attributes for JPC-derived secretome therapeutics [49,50]. Passage number alone should not be regarded as a universal release criterion because expansion rates, cumulative population doublings, donor characteristics, and manufacturing conditions differ among laboratories [51,52]. Monitoring source-cell metabolic activities/viability, senescence-associated markers, conditioning conditions, secretome composition, and functional bioactivity may provide a more robust framework for ensuring batch-to-batch consistency and therapeutic efficacy [49,50,53]. Accordingly, the comparison between P5 and P10 should be interpreted as demonstrating that source-cell expansion state can substantially influence product quality under the applied manufacturing conditions rather than defining a universal passage threshold for clinical production.

Several limitations should be acknowledged. First, as the causal contribution of individual secretome components was not investigated, mechanistic validation using neutralization or recombinant supplementation approaches will be necessary to identify key potency-mediating factors. Second, although NGN2-induced neurons provide a robust and standardized neuronal model, they do not fully reproduce the complexity of sensory or motor neurons relevant to craniofacial nerve repair or the multicellular environment of the injured neural niche. Third, donor-dependent variability was not evaluated because secretomes from four donors were pooled before proteomic analysis. Consequently, the four proteomic measurements per passage represented technical replicates of a single pooled-secretome preparation, and the corresponding adjusted *p* values reflect technical rather than inter-donor biological variability. The proteomic findings should therefore be interpreted as an exploratory comparison of the pooled P5 and P10 preparations. Fourth, we did not assess cumulative population-doubling data to precisely define source-cell expansion history, and equal protein dosing standardized the amount of administered secretome but did not account for secretome productivity per viable source cell, and dose–response relationships were not examined. Finally, neuronal assays were performed using a single NGN2 transduction batch across 3 repeated thaw-and-treatment experiments, thereby assessing experimental reproducibility rather than neuronal donor or differentiation-batch variability. Future studies should therefore incorporate cumulative population-doubling analyses, mechanistic validation of candidate potency markers, dose–response studies, and more physiologically relevant neural models to support the establishment and validation of critical quality attributes for clinical-grade JPC-derived secretome products.

## 5. Conclusions

The present study demonstrates that prolonged in vitro expansion induces a senescence-like phenotype in JPCs, remodels the composition of JPC-derived secretomes, and diminishes their neuro-supportive potency in vitro. Early-passage secretomes more effectively promoted neuronal survival and neurite outgrowth, whereas late-passage secretomes exhibited a shift toward inflammatory and stress-associated signaling. Collectively, these findings identify the expansion state of the producer cells and the resulting secretome signature as candidate critical quality attributes that should be considered during the manufacturing, quality control, and standardization of JPC-derived secretome therapeutics for neural regeneration.

## Figures and Tables

**Figure A1:**
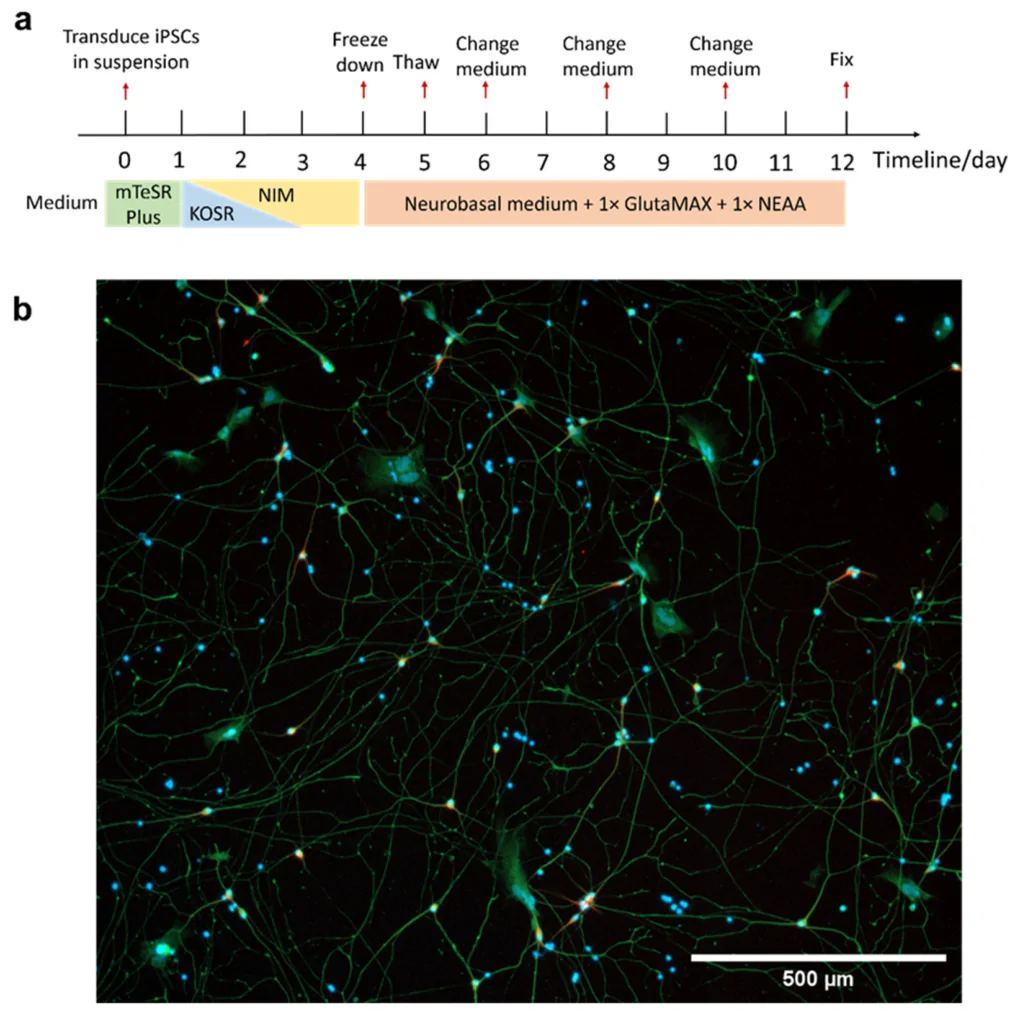
(**a**) Timeline of NGN2-induced neuronal induction. Schematic overview of the stepwise differentiation protocol, showing the sequential culture media conditions during NGN2-induced neuronal induction. Red arrows indicate key intervention time points. Abbreviations: mTeSR Plus, mTeSR™ Plus medium; KOSR, KnockOut™ Serum Replacement medium; NIM, neuronal induction medium; NEAA, non-essential amino acids. (**b**) Representative immunofluorescence image of NGN2-induced neurons on day 12, showing neuronal marker expression and extensive neurite outgrowth. Cells were stained for MAP2 (red) and doublecortin (green), with nuclei counterstained with DAPI (blue). Scale bar, 500 μm.

## Data Availability

The mass spectrometry proteomics data generated in this study have been deposited with the ProteomeXchange Consortium under the dataset identifier PXD082410.

## Supplementary Materials

The following supporting information can be downloaded at: https://www.mdpi.com/article/10.3390/cells15171526/s1, Table S1: Complete proteomic dataset analyzed in this study, including differentially abundant proteins and functional enrichment results.

## Appendix A

### Appendix A.1. Trilineage Differentiation Assays

For osteogenic differentiation, 4 × 10^4^ JPCs were seeded into 6-well plates pre-coated with 0.1% gelatin (Sigma-Aldrich, St. Louis, MO, USA). Cells were cultured for 21 days in DMEM/F-12 (Gibco, Thermo Fisher Scientific, Waltham, MA, USA) containing 10% human platelet lysate (hPL; PL BioScience, Aachen, Germany), 100 U/mL penicillin-streptomycin (P/S; Gibco), and 2.5 µg/mL amphotericin B (Neofroxx, Einhausen, Germany), 100 µM L-ascorbic acid 2-phosphate (Sigma-Aldrich), 10 mM β-glycerophosphate (AppliChem, Darmstadt, Germany), and 4 µM dexamethasone (Sigma-Aldrich), with medium changes every 2–3 days. Osteogenic differentiation was confirmed by Alizarin Red staining.

For adipogenic differentiation, JPCs were seeded at 2 × 10^4^ cells/cm^2^ onto 0.1% gelatin-coated culture dishes. After reaching confluence, cells were cultured for 21 days using alternating cycles of adipogenic induction medium A for 3 days and maintenance medium B for 1 day. Medium A consisted of DMEM/F-12 supplemented with 10% fetal bovine serum (FBS; Sigma-Aldrich), 100 U/mL P/S, 2.5 µg/mL amphotericin B, 1 µM dexamethasone, 0.2 mM indomethacin (Sigma-Aldrich), 10 µg/mL insulin (Gibco), and 0.5 mM 3-isobutyl-1-methylxanthine (IBMX; Sigma-Aldrich), whereas medium B contained the same basal medium without dexamethasone, indomethacin, or IBMX. Adipogenic differentiation was assessed by Oil Red O staining.

For chondrogenic differentiation, 2.5 × 10^5^ JPCs in 200 µL were pelleted in 96-well V-bottom polypropylene plates by centrifugation at 350× *g* for 5 min and cultured for 21 days in high-glucose DMEM (Gibco, Thermo Fisher Scientific, Waltham, MA, USA) containing 100 U/mL P/S, 2.5 µg/mL amphotericin B, 1× ITS+ Premix (Corning Inc., Corning, NY, USA), 1 µM L-ascorbic acid 2-phosphate, 0.1 µM dexamethasone, and 10 ng/mL recombinant human TGF-β1 (PeproTech, Rocky Hill, NJ, USA). The medium was changed every 3 days. Chondrogenic differentiation was verified by Toluidine Blue staining.

### Appendix A.2. NGN2-Induced Neuronal Induction

NGN2-induced neurons, generated by doxycycline-induced expression of the proneural transcription factor neurogenin-2 (NGN2/NEUROG2), were obtained from human JPC-derived iPSCs using a modified protocol (Appendix A, Figure A1). Lentiviral vectors pLV-TetO-hNGN2-GFP-Puro and FUdeltaGW-rtTA were obtained from the collaborating laboratory of Dr. Julia Fitzgerald, and added at 0.25 µL/mL each, resulting in final particle concentrations of approximately 1.22 × 10^8^ and 1.75 × 10^8^ lentiviral particles/mL, respectively, in the transduction mixture.

On day 0, approximately 6 × 10^6^ iPSCs were dissociated with Accutase (Thermo Fisher Scientific, Waltham, MA, USA), resuspended in 1.2 mL viral suspension containing 10 µM Y-27632 (Rock-inhibitor, Selleck Chemicals), and incubated for 5 min at room temperature. The transduction mixture (200 µL/well) was pipetted onto Matrigel (Corning Inc.)-coated 6-well plates, pre-equilibrated with 800 µL mTeSR Plus medium (STEMCELL Technologies, Vancouver, BC, Canada), and supplemented with 10 µM Y-27632. After 24 h, the medium was replaced with KOSR medium composed of KnockOut™ DMEM, 15% KnockOut™ Serum Replacement, 1× GlutaMAX, and 1× non-essential amino acids (NEAA) (all from Gibco), 10 µM SB431542 (TGF-β/Smad inhibitor, Selleck Chemicals), 2 µM XAV939 (inhibitor of Wnt signaling, Selleck Chemicals), 100 nM LDN-193189 (inhibitor of BMP signaling, STEMCELL Technologies), and 0.1% 2-mercaptoethanol (Sigma-Aldrich). Doxycycline (1 µg/mL) (Sigma-Aldrich) was added to induce NGN2 expression.

On day 2, the medium was changed to a 1:1 mixture of KOSR medium and neural induction medium (NIM), supplemented with doxycycline (1 µg/mL) and puromycin (1 µg/mL; Sigma-Aldrich). NIM consisted of DMEM/F-12 medium supplemented with 1× GlutaMAX, 1× NEAA, 1× N2 supplement (Gibco), 3 g/L glucose (Gibco), and 2 µg/mL heparin (STEMCELL Technologies). On day 3, cultures were maintained in NIM containing 1 µg/mL doxycycline and 1 µg/mL puromycin to continue selection.

On day 4, cells were transferred to neuronal medium consisting of Neurobasal medium (Gibco) supplemented with 1× GlutaMAX, 1× NEAA, 1× N2 supplement, 1× B-27 Supplement (Gibco), 10 ng/mL BDNF, and 1 µg/mL doxycycline. At this stage, NGN2-induced neurons were either used for downstream applications or cryopreserved in neuronal medium containing 10 µM Y-27632 and 10% dimethyl sulfoxide (DMSO; Sigma-Aldrich). A single NGN2 transduction batch was generated and 3 independently performed thaw-and-treatment series were conducted for the secretome experiments. Pooled secretomes were added to 3–4 replicates of each thaw-and-treatment experiment, so 9–10 wells in total were visualized and quantified for each condition.

**Table A1:** Primer sequences.

Gene	Sequence P1	Sequence P2
GAPDH	ACA TCG CTC AGA CAC CATG	TGT AGT TGA GGT CAA TGA AGGG
IL6	ATT CGT TCT GAA GAG GTG AGTG	CCT TCC CTG CCC CAG TA
IL8	GAG ACA GCA GAG CAC ACA AG	CTT CAC ACA GAG CTG CAG AA
CCL2	AGC AGC CAC CTT CAT TCC	GCC TCT GCA CTG AGA TCT TC
CCL20	CCA TGT GCT GTA CCA AGA GT	TTA GGA TGA AGA ATA CGG TCT GTG
VEGF-A	CCA TGA ACT TTC TGC TGT CTTG	GCG CTG ATA GAC ATC CAT GA
TGFβ1	CCG ACT ACT ACG CCA AGGA	GTT CAG GTA CCG CTT CTCG
p16	AGC TGT CGA CTT CAT GAC AAG	TGA GCT TTG GTT CTG CCA TT
p21	GCA GAC CAG CAT GAC AGAT	GAG ACT AAG GCA GAA GAT GTA GAG
p53	TGTGACTTGCACGTACTCCC	ACCATCGCTATCTGAGCAGC
βNGF	CGG ACC CAA TAA CAG TTT TACC	TGT GAT CAG AGT GTA GAA CAA CA
GDNF	TCA TAG TAA CAA GAA GCA GCA GT	GAG CAG AAA GGA CAG AGA AGG

**Table A2:** Genes corresponding to strongly differentially abundant secretome proteins between JPC-P5 and JPC-P10 secretomes (adjusted *p*-value < 0.05 and |log2FC| ≥ 4). GO BP terms represent enriched Gene Ontology Biological Process annotations associated with the listed genes. Mean protein abundance represents the normalized mean protein abundance across technical replicates. Abbreviations: JPC P5, jaw periosteum cells at passage 5; JPC P10, jaw periosteum cells at passage 10; GO BP, Gene Ontology Biological Process; log2FC, log2 fold change. NA indicates that no related GO BP terms were identified in the GO enrichment analysis.

Genes	Full Name	Enriched GO BP Terms	Mean Protein Abundance		Adjusted *p* Value
			JPC P5	JPC P10	
SYT1	Synaptotagmin 1	regulation of cell morphogenesis; cell growth; response to metal ion; regulation of cell growth; developmental growth involved in morphogenesis; developmental cell growth	15.67 ± 0.35	9.15 ± 0.79	0.005
S100A4	S100 calcium binding protein A4	mesenchymal cell differentiation; mesenchyme development; chemotaxis; taxis	15.25 ± 1.44	10.15 ± 1.70	0.014
CCDC148	Coiled-coil domain containing 148	NA	15.05 ± 1.21	10.61 ± 2.53	0.048
MCM3AP	Minichromosome maintenance complex component 3 associated protein	nucleocytoplasmic transport; nuclear transport; RNA export from nucleus; mRNA transport; nuclear export; mRNA export from nucleus; nucleic acid transport; RNA transport; establishment of RNA localization; nucleobase-containing compound transport	13.62 ± 0.35	9.25 ± 0.36	0.007
HHIP	Hedgehog interacting protein	lung development; respiratory tube development; respiratory system development; morphogenesis of a branching structure; branching morphogenesis of an epithelial tube; morphogenesis of a branching epithelium; epithelial tube branching involved in lung morphogenesis	14.05 ± 0.18	9.80 ± 1.11	0.005
HAS1	Hyaluronan synthase 1	ameboidal-type cell migration; extracellular matrix organization; extracellular structure organization; external encapsulating structure organization	13.45 ± 2.52	9.31 ± 0.19	0.044
EIF2AK1	Eukaryotic translation initiation factor 2 alpha kinase 1	multicellular organismal-level chemical homeostasis	13.58 ± 0.26	9.49 ± 0.70	0.004
SOD3	Superoxide dismutase 3	response to metal ion; reactive oxygen species metabolic process; response to oxygen levels; response to decreased oxygen levels; response to hypoxia; response to copper ion; cellular response to oxidative stress; response to oxidative stress	10.06 ± 0.40	14.07 ± 0.36	0.004
LAMA1	Laminin subunit alpha 1	positive regulation of cell adhesion; lung development; respiratory tube development; respiratory system development; morphogenesis of a branching structure; branching morphogenesis of an epithelial tube; morphogenesis of a branching epithelium; extracellular matrix organization; extracellular structure organization; external encapsulating structure organization; gland morphogenesis; epithelial tube branching involved in lung morphogenesis	9.66 ± 0.57	13.80 ± 0.06	0.006
ISG20	Interferon stimulated exonuclease gene 20	defense response to virus; response to virus; negative regulation of viral genome replication; negative regulation of viral process; regulation of viral genome replication; regulation of viral life cycle; regulation of viral process; viral genome replication; viral process; viral life cycle	9.93 ± 0.49	14.08 ± 1.79	0.016
IL17B	Interleukin 17B	positive regulation of cytokine production	9.88 ± 0.69	14.05 ± 0.83	0.005
NAPSA	Napsin A aspartic peptidase	surfactant homeostasis; multicellular organismal-level chemical homeostasis	9.84 ± 0.58	14.02 ± 1.51	0.010
CLYBL	Citrate lyase beta like	vitamin metabolic process; water-soluble vitamin metabolic process	10.17 ± 1.21	14.38 ± 0.20	0.006
HOGA1	4-hydroxy-2-oxoglutarate aldolase 1	small molecule catabolic process; cellular modified amino acid metabolic process; organic acid catabolic process; carboxylic acid catabolic process; dicarboxylic acid metabolic process; carboxylic acid biosynthetic process; organic acid biosynthetic process; alpha-amino acid catabolic process; carbohydrate derivative catabolic process; amino acid catabolic process	10.84 ± 0.38	15.23 ± 0.63	0.004
TNFAIP6	Tumor necrosis factor alpha-induced protein 6	negative regulation of response to external stimulus; ossification; regulation of chemotaxis; negative regulation of chemotaxis; chemotaxis; taxis; osteoblast differentiation	9.60 ± 0.45	14.00 ± 0.54	0.004
IFI44	Interferon-induced protein 44	response to virus	10.79 ± 1.38	15.33 ± 0.45	0.006
IFI44L	Interferon-induced protein 44 like	defense response to virus; response to virus	10.25 ± 1.23	14.90 ± 0.36	0.006
ALDH1A1	Aldehyde dehydrogenase 1 family member A1	carboxylic acid biosynthetic process; organic acid biosynthetic process; carbohydrate derivative catabolic process	10.06 ± 0.88	14.71 ± 0.51	0.004
OAS2	2′-5′-oligoadenylate synthetase 2	defense response to virus; response to virus; negative regulation of viral genome replication; negative regulation of viral process; regulation of viral genome replication; regulation of viral life cycle; regulation of viral process; positive regulation of type I interferon production; regulation of type I interferon production; type I interferon production; response to type I interferon; positive regulation of interferon-beta production; viral genome replication; cellular response to type I interferon; interferon-mediated signaling pathway; interferon-beta production; regulation of interferon-beta production; type I interferon-mediated signaling pathway; viral process; viral life cycle; positive regulation of cytokine production	9.91 ± 0.94	14.66 ± 0.47	0.003
USP18	Ubiquitin-specific peptidase 18	defense response to virus; response to virus; negative regulation of innate immune response; antiviral innate immune response; negative regulation of response to external stimulus; response to type I interferon; regulation of innate immune response; negative regulation of response to biotic stimulus; cellular response to type I interferon; interferon-mediated signaling pathway; type I interferon-mediated signaling pathway; negative regulation of type I interferon-mediated signaling pathway; regulation of type I interferon-mediated signaling pathway; regulation of response to cytokine stimulus	9.40 ± 0.61	14.28 ± 1.65	0.008
PAPLN	Papilin, proteoglycan-like sulfated glycoprotein	extracellular matrix organization; extracellular structure organization; external encapsulating structure organization	9.48 ± 0.52	14.37 ± 0.23	0.006
SLC15A3	Solute carrier family 15 member 3	regulation of innate immune response; cytoplasmic pattern recognition receptor signaling pathway; pattern recognition receptor signaling pathway; positive regulation of innate immune response; innate immune response-activating signaling pathway	10.19 ± 0.45	15.10 ± 1.86	0.010
CDH16	Cadherin 16	NA	9.67 ± 0.79	14.70 ± 0.79	0.004
FZD3	Frizzled class receptor 3	response to xenobiotic stimulus; regulation of nervous system development; regulation of neurogenesis; positive regulation of nervous system development; axon guidance; neuron projection guidance	9.76 ± 1.24	14.80 ± 1.75	0.013
RARRES2	Retinoic acid receptor responder 2	regulation of chemotaxis; chemotaxis; taxis	9.43 ± 0.61	14.65 ± 0.86	0.004
MX1	MX dynamin-like GTPase 1	defense response to virus; response to virus; negative regulation of viral genome replication; negative regulation of viral process; regulation of viral genome replication; regulation of viral life cycle; regulation of viral process; antiviral innate immune response; response to type I interferon; viral genome replication; viral process; viral life cycle	14.38 ± 1.65	19.74 ± 0.35	0.006
RNF17	Ring finger protein 17	NA	12.27 ± 1.71	18.13 ± 1.36	0.009
ENPEP	Glutamyl aminopeptidase	NA	8.94 ± 0.68	14.95 ± 0.33	0.007
SERPING1	Serpin family G member 1	negative regulation of innate immune response; negative regulation of response to external stimulus; regulation of innate immune response; negative regulation of response to biotic stimulus; regulation of endopeptidase activity; wound healing	9.35 ± 0.14	15.60 ± 0.88	0.005
